# The Role of Hemoglobin in Temporomandibular Joint Osteoarthritis and the Therapeutic Potential of Hydroxyurea

**DOI:** 10.1155/ijod/3607127

**Published:** 2026-05-19

**Authors:** Min Hui, Zhihua Xu, Qinghua Li, Ying Zhan, Yuqian Shi, Fan Wu, Lei Lu, Mian Zhang, Hongxu Yang, Shibin Yu, Jing Zhang

**Affiliations:** ^1^ School of Medicine, Northwest University, Xi’an, Shaanxi, China, nwu.edu.cn; ^2^ State Key Laboratory of Oral and Maxillofacial Reconstruction and Regeneration, National Clinical Research Center for Oral Diseases, Shaanxi International Joint Research Center for Oral Diseases, Department of Oral Anatomy and Physiology, School of Stomatology, Fourth Military Medical University, Xi’an, 710032, Shaanxi, China, fmmu.edu.cn

**Keywords:** cartilage, condyle, hemoglobin, hydroxyurea, osteoarthritis, temporomandibular joint

## Abstract

**Background:**

Temporomandibular joint osteoarthritis (TMJ OA) is characterized by articular cartilage degeneration involving chondrocyte loss and matrix degradation. However, the underlying mechanisms remain elusive. Hemoglobin (HB) has been identified in chondrocytes and is closely associated with cell survival.

**Objective:**

This study aims to investigate the role of HB in condylar cartilage degeneration during the early stage of TMJ OA and evaluate the potential protective effects of hydroxyurea (HU), a therapeutic agent used for sickle cell disease, suggesting its promise as a novel therapeutic strategy against OA.

**Methods:**

The TMJ OA model was established in 6‐week‐old female rats using unilateral anterior crossbite (UAC). ATDC5 chondrocytes were exposed to fluid flow shear stress (FFSS) to mimic OA‐like changes in vitro. Histological and immunohistochemical staining, real‐time PCR, and Western blot analysis were performed to detect the morphological and molecular changes in condylar cartilage.

**Results:**

The results showed a significant downregulation of *HBA* and *HBB* alongside cartilage degeneration in the UAC group, characterized by decreased cartilage thickness, degraded cartilage matrix, and increased matrix metalloproteinases (MMPs). FFSS‐stimulated chondrocytes also exhibited decreased *HBA* and *HBB* expression. Notably, HU treatment enhanced γ‐globin gene (*HBG*) expression, mitigated FFSS‐stimulated chondrocyte degeneration in vitro, and attenuated TMJ OA progression in vivo. Specifically, HU ameliorated the degenerative phenotype in cartilage, increasing matrix content and reducing matrix‐degrading enzyme expression.

**Conclusions:**

These findings identify HB subunits as novel molecular targets for OA therapy, with HU demonstrating significant chondroprotective efficacy. The study provides mechanistic insights into OA pathogenesis and highlights a promising repurposing therapeutic strategy for clinical intervention.

## 1. Introduction

Temporomandibular joint osteoarthritis (TMJ OA), one of the severe clinical phenotypes within TMJ disorders, is pathologically characterized by articular cartilage degeneration and abnormal subchondral bone remodeling [1]. In advanced stages, TMJ OA significantly impairs orofacial functions, including mastication, speech, and facial expression, substantially compromising patients’ quality of life, physical well‐being, and psychological health [2]. The degenerative process of OA cartilage is characterized by chondrocyte apoptosis, extracellular matrix (ECM) degradation, and progressive thinning of the articular cartilage layer. Despite considerable research advances, the molecular and cellular mechanisms underlying these pathological changes remain incompletely understood, necessitating further investigation [3].

Hemoglobin (HB), an iron‐protoporphyrin‐based tetrameric metalloprotein, comprises four heme groups, each bound to a polypeptide chain. These polypeptide chains form two distinct pairs of globin subunits: α‐globin and β‐globin chains. Predominantly localized in erythrocytes, HB functions as an essential oxygen‐transport molecule, mediating reversible binding of oxygen in pulmonary capillaries and subsequent release in peripheral tissues [4]. However, emerging research over recent decades has revealed ectopic HB expression in various non‐erythroid somatic cells. This abnormal expression has been implicated in the pathogenesis of multiple human diseases, suggesting HB’s potential role in disease‐specific pathophysiological mechanisms [5–10].

Recent investigations have identified HB body (Hedy) in chondrocytes, mediating oxygen delivery to chondrocytes and suggesting a critical association with chondrocyte viability and survival [11]. Clinical studies have further revealed a significant correlation between anemia and joint pathologies. In patients with rheumatoid arthritis, HB levels are independently associated with disease activity, with lower HB levels observed in those with higher disease activity [12, 13]. Furthermore, patients with thalassemia, a condition characterized by chronic anemia, exhibit a high prevalence of bone disease, including osteoporosis and fractures [14]. Although these findings do not establish causality, they suggest a potential link between anemia and joint/bone pathology.

We hypothesize that HB also plays a significant role in cartilage degeneration during TMJ OA pathogenesis. To test this hypothesis, we used a unilateral anterior crossbite (UAC) rodent model that recapitulates TMJ OA‐like pathological changes [15, 16]. In parallel, an in vitro OA model was created by applying fluid flow shear stress (FFSS) stimulation to ATDC5 chondrocytes. Hydroxyurea (HU) is a US Food and Drug Administration (FDA)‐approved antimetabolite chemotherapeutic agent primarily used to treat sickle cell disease and chronic myeloid leukemia. In sickle cell disease, HU exerts its therapeutic effect by upregulating γ‐globin gene (*HBG*) expression, promoting fetal HB (HBF) formation via γ‐globin and α‐globin binding [17–19]. This study aims to elucidate the mechanistic role of HB in cartilage degeneration and the therapeutic potential of HU during TMJ OA progression.

## 2. Materials and Methods

### 2.1. Animals

This study was conducted in accordance with the ARRIVE 2.0 guidelines for reporting in vivo experiments. All animal experiments were approved by the Animal Ethics Committee of the School of Stomatology (2024‐005). Based on our previously published studies using the UAC model [16], we determined that a minimum of six rats per group would be sufficient to detect differences in the primary outcome (cartilage thickness) with adequate statistical power (80%) at a two‐tailed significance level of *α* = 0.05. To perform three independent assays (histology, Western blot, and real‐time PCR), each requiring six independent samples, we initially included nine rats per group. This provided 18 condyles to allocate six condyles for histology, six for Western blot, and six for real‐time PCR, ensuring a final sample size of *n* = 6 per assay.

Forty‐five 6‐week‐old female Sprague‐Dawley rats (weight 140–160 g) were purchased from the Animal Center of Fourth Military Medical University (FMMU). All animal care procedures were approved by the Administration Committee of Experimental Animals at the FMMU and performed according to the Institutional Animal Care Guidelines. Female rats were used for two reasons. First, temporomandibular disorders (TMDs), including TMJ OA, have a well‐documented higher prevalence in females than in males [20]; thus, using female rats enhances the clinical relevance of our findings. Second, using a single sex avoids the potential confounding effects of hormonal variations between sexes in this exploratory study. The estrus cycle was not monitored, which we acknowledge as a limitation. The rats were randomly divided into five subgroups of nine rats each: 1‐week control, 1‐week UAC, 4‐week control, 4‐week UAC, and 4‐week UAC + HU. Randomization was performed by assigning each rat a unique ID number and sorting them according to a computer‐generated random number sequence. Animals were allocated to experimental groups in blocks of nine consecutive rats from the sorted list, with each group subsequently housed three rats per cage (three cages per experimental group). No animals or data points were excluded from any experimental groups in this study.

### 2.2. UAC Modeling

UAC was established as previously reported by our group. The method was originally developed in mice by Lu et al. [15] and subsequently adapted for rats by Zhang et al. [16]. The maxillary aberrant prosthesis was made of 25# needle (Shinva Ande, Shandong, China; length = 2.5 mm, inner diameter = 3 mm), and the mandibular prosthesis was made of 20# needle with a 135° inclined guide plate (length = 4.5 mm and inner diameter = 3.5 mm). Prior to the procedure, rats were deeply anesthetized using isoflurane. The prostheses were then bonded to the left incisors to create a crossbite. Rats in the control groups received identical procedures excluding prosthesis bonding.

### 2.3. TMJ Injection

Intra‐articular injections of HU (50 μL per side, 25 μmol/mL; HY‐B0313, MedChemExpress, NJ, USA) were administered twice weekly for 4 weeks, starting 3 days after the UAC operation until the animals were euthanized. Rats in the UAC‐only and control groups received intra‐articular injections of an equal volume (50 μL) of sterile saline on the same schedule to control for the injection procedure. Under deep anesthesia with isoflurane, rats were placed in lateral recumbency. A microinjector was inserted inferior to the zygomatic arch until contacting the mandibular ramus, then advanced along the osseous surface into the TMJ space.

The timing of injections (3 days post‐UAC) was chosen to allow for the establishment of initial OA‐like changes prior to intervention, thereby simulating a therapeutic rather than a preventive approach. The concentration (25 μmol/mL) and dosing regimen (twice weekly for 4 weeks) were selected based on three considerations: (1) preliminary dose–response experiments in ATDC5 cells demonstrated that 25 μmol/mL effectively upregulated HBG expression without compromising cell viability (Figure S1); (2) previous studies have shown that HU at concentrations ranging from 25 to 100 μmol/L effectively induces HB expression in other cell types [21, 22]; and (3) the twice‐weekly regimen was chosen based on the prolonged pharmacodynamic effect of HU on HB induction despite its short half‐life [23, 24], allowing for sustained therapeutic effect while minimizing stress from repeated injections.

### 2.4. Tissue Preparation

No significant differences were observed in the histomorphology and molecular properties between left and right TMJs in UAC rats [16]. From each group of nine rats, the right condyles from six rats were used for immunohistochemistry, the left condyles from another six rats were used for Western blot analysis, and the remaining three left and three right condyles were used for quantitative real‐time PCR, providing six independent samples per assay. For histochemistry and immunohistochemistry staining, TMJ tissue blocks were fixed with 4% paraformaldehyde at 4°C for 12 h, followed by decalcification in 4% ethylene‐diaminetetraacetic acid disodium salt (EDTA‐2Na) solution for 6 weeks at room temperature. The tissues were then dehydrated in graded alcohol solutions and embedded in paraffin. For real‐time PCR and Western blot analyses, the condylar cartilages were preserved at −80°C for RNA and protein extraction.

### 2.5. Histological Staining and Analysis

Five‐μm sagittal sections were stained with hematoxylin and eosin (H&E) and Safranin O (SO). Images were acquired using a Leica DM 2500 optical microscope (Wetzlar, Germany). To evaluate the histomorphological changes during TMJ OA progression, condylar cartilage images were divided into three sections of equal width (anterior, middle, and posterior). A region of interest (ROI) centered in each section was delineated (width: 200 μm, height: equal to cartilage thickness) and analyzed for cartilage thickness and SO‐positive area. Histological analysis was performed independently by two investigators blinded to group allocation, with the mean value calculated from their assessments.

### 2.6. Immunohistochemical Staining and Analysis

The sections were dewaxed, followed by hydrogen peroxide blocking, antigen retrieval, and serum blocking. Subsequently, the sections were incubated overnight at 4°C with primary antibodies: HBA (1:50, 14537‐1‐AP, Proteintech, IL, USA), HBB (1:50, 16216‐1‐AP, Proteintech, IL, USA), and type II collagen (COL II) antibody (1:200, M2139, Santa Cruz Biotechnology, CA, USA). A two‐step detection kit (PV‐9001, Zhongshan Golden Bridge, Beijing, China) was used for secondary antibody incubation, and DAB chromogen (ZLI‐9018, Zhongshan Golden Bridge, Beijing, China) was applied for signal visualization. Cells were considered HBA‐ or HBB‐positive if they exhibited distinct brown cytoplasmic staining with intensity above the background level observed in negative control sections (where the primary antibody was omitted). Chondrocytes were specifically identified by their characteristic rounded morphology and location within the cartilage lacunae in the condylar cartilage sections. The percentage of HBA‐ and HBB‐positive cells was calculated by dividing the number of positively stained chondrocytes by the total number of chondrocytes within the ROI in the center third of the cartilage, then multiplying by 100. Immunohistochemical staining results were independently analyzed by two investigators blinded to group allocation, with the mean value derived from their assessments.

### 2.7. FFSS

The mouse ATDC5 chondrogenic cell line (iCell‐m804; Cellverse, Shanghai, China) was utilized for in vitro experiments. ATDC5 is a well‐characterized chondrogenic cell line derived from mouse teratocarcinoma AT805 [25, 26]. This cell line has been extensively used as an in vitro model for studying chondrocyte biology, cartilage ECM synthesis, and OA pathogenesis [27]. Compared to primary chondrocytes, ATDC5 cells offer greater stability and proliferative capacity, providing a consistent and reproducible system for mechanical stimulation studies. The ATDC5 chondrocytes were seeded onto culture plates in DMEM supplemented with 10% fetal bovine serum and 1% penicillin‐streptomycin. The plates were cultured in a 37°C, hypoxic conditions (5% O_2_ and 5% CO_2_) incubator and allowed to adhere for 24 h. The Flexcell Streamer system was used to apply a shear stress of 16 dyn/cm^2^ to ATDC5 cells for different durations. In our experiments, shear stress was applied for 1 and 2 h, and cells were collected immediately after the application to extract RNA and proteins for analysis.

### 2.8. Quantitative Real‐Time PCR

Total RNA and proteins were extracted from rat condylar cartilage and in vitro‐cultured ATDC5 cells using Tripure (Cat. No. 11667165001, Roche, Basel, Switzerland). First‐strand cDNA synthesis was performed with MightyScript Master mix (Cat. No.B639251‐0100, BBI, Shanghai, China), according to the manufacturer’s protocol. The expression of *COL2*, matrix metalloproteinase 3 (*MMP3*), *MMP9*, *MMP13*, and *HBG* was determined by quantitative real‐time PCR (qRT‐PCR) using a CFX Connect Real‐Time System (Bio‐Rad, CA, USA) with SGExcel FastSYBR qRT‐PCR Premix (Cat. No. B639271‐0005, BBI, Shanghai, China,), using cDNA as a template. The primers are listed in Table S1.

### 2.9. Western Blot

The total protein was separated by SDS‐PAGE for 90 min. Proteins were transferred onto a PVDF membrane using transfer buffer for 1 h. The membrane was then blocked for 1 h at 37°C with quick blocking solution (Cat. No. CW0054M, CWBIO, Jiangsu, China). The membrane was incubated overnight at 4°C with primary antibodies: anti‐HBA (1:2000, Cat. No. ab92492, 1:2000, Abcam, UK), anti‐HBB (1:2000, Cat. No. ab214049, Abcam, UK), anti‐β‐actin (1:10000, Cat. No. 66009‐1‐Ig, Proteintech, IL, USA), and HBG (1:2000, orb627618, Biorbyt, Cambridge, UK). Subsequently, the membrane was incubated for 1 h at 37°C with species‐matched HRP‐conjugated secondary antibodies: Goat Anti‐Rabbit IgG (1:10000, Cat. No. RGAR001, Proteintech, IL, USA) and Goat Anti‐Mouse IgG (1:2000, Cat. No. SA00001‐1, Proteintech, IL, USA). Protein bands were visualized using enhanced chemiluminescence (Cat. No. RM00021P, Abclone, Wuhan, China) and analyzed with Image Lab software (v5.1, Bio‐Rad, CA, USA). Finally, protein levels were quantified and normalized to β‐actin expression.

### 2.10. Statistical Analysis

Statistical analyses were performed using GraphPad Prism 9.0. For animal experiments, data were analyzed using independent samples (*n* = 6 rats per group). For in vitro experiments, data were derived from three independent experiments (*n* = 3), each performed in triplicate. Data were presented as mean ± SD. Normality and homogeneity of variances were assessed with Shapiro–Wilk and Levene’s tests, respectively. Nonparametric tests (Mann–Whitney *U* or Kruskal–Wallis with Dunn’s post hoc test) were employed as the primary analysis. Mean differences with 95% confidence intervals are reported alongside two‐tailed *p*‐values, with *p*  < 0.05 considered significant.

## 3. Results

### 3.1. UAC Induced Pathological Changes of OA in Rat Condylar Cartilage

**Figure 1:**
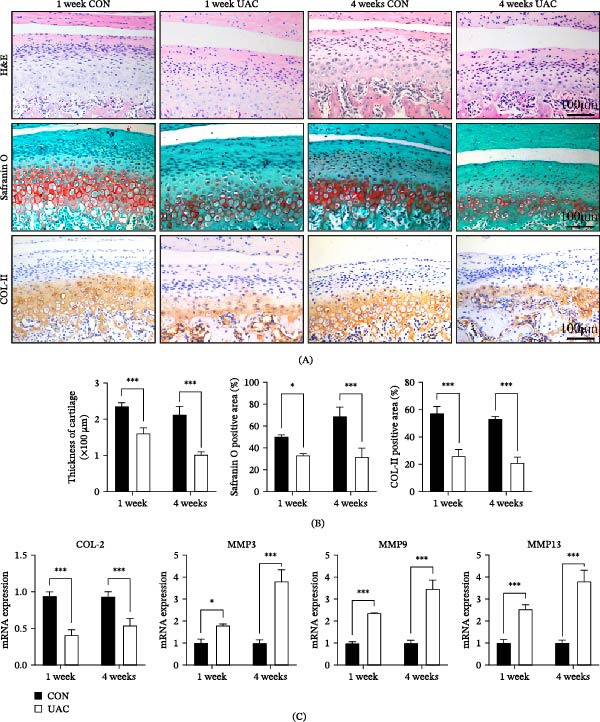
Degeneration of condylar cartilage in rats with temporomandibular joint osteoarthritis (TMJ OA) induced by unilateral anterior crossbite (UAC) stimulation. (A, B) Representative images of hematoxylin and eosin (H&E) staining showing significantly decreased cartilage thickness in UAC rats. Safranin O (SO) staining reveals markedly reduced proteoglycan content in UAC rats. Immunohistochemistry (IHC) staining demonstrates significant reduction of type II collagen (COL II) in UAC rats. (C) mRNA expression of *COL2* was decreased in UAC rats, while matrix metalloproteinase 3 (*MMP3*), *MMP9*, and *MMP13* were increased. Data are presented as mean ± SD (*n* = 6 rats per group). CON, control;  ^∗^ *p* < 0.05;  ^∗∗^ *p* < 0.01;  ^∗∗∗^ *p* < 0.001, scale bars: 100 μm.

In this experiment, 6‐week‐old female Sprague‐Dawley rats were used to establish a TMJ OA model through UAC treatment. Condylar cartilage samples were collected at 1 and 4 weeks after modeling for morphological and molecular analyses. H&E staining of paraffin sections revealed significant histomorphometric alterations in UAC‐treated rats. Compared to the control group, the articular cartilage exhibited reduced thickness (both time points: *p* < 0.001) and disorganized chondrocyte layers, indicative of degenerative changes (Figure 1A, B). SO staining and COL II immunohistochemistry showed statistically significant decreases in proteoglycan (1 week: *p*  < 0.05; 4 weeks: *p* < 0.001) and collagen content (both time points: *p* < 0.001), demonstrating impaired ECM composition (Figure 1A, B). Quantitative real‐time PCR analysis revealed decreased mRNA expression of *COL2* (both time points: *p* < 0.001), alongside increased expression of *MMP3* (1 week: *p* < 0.05; 4 weeks: *p* < 0.001), *MMP9*, and *MMP13* (both time points: *p* < 0.001) (Figure 1C). These results confirm that UAC stimulation successfully induced TMJ OA‐like degenerative changes in rat condylar cartilage.

### 3.2. HB Expression was Decreased in UAC Induced Degenerative Condylar Cartilage

**Figure 2:**
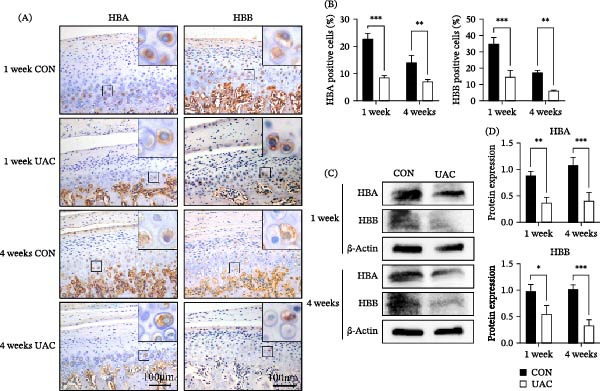
Hemoglobin expression was significantly reduced in the condylar cartilage of TMJ OA rats. (A, B) IHC straining showed that the percentages of HBA positive cells and HBB positive cells were significantly decreased in TMJ OA rats. Higher magnification images of the boxed areas are shown as insets to better visualize positively stained chondrocytes (brown cytoplasmic staining). Quantification of the percentage of HBA positive and HBB positive cells is shown in the right panels. (C, D) Western blot analysis quantified HBA (~15 kDa), HBB (~16 kDa), and β‐actin (~42 kDa) protein levels in total protein lysates extracted from chondrocytes of the 1‐week and 4‐week UAC groups and their corresponding control groups. Full, uncropped Western blot images are provided in Figures S2 and S3. Quantification of HBA and HBB protein levels normalized to β‐actin. Data are presented as mean ± SD (*n* = 6 rats per group). CON, control; UAC, unilateral anterior crossbite;  ^∗^ *p* < 0.05;  ^∗∗^ *p* < 0.01;  ^∗∗∗^ *p* < 0.001, scale bars: 100 μm.

As shown in Figure 2A, B, immunohistochemical staining revealed that HB expression levels were significantly reduced in UAC‐treated condylar cartilage compared to the control group. This was demonstrated by the decreased percentages of HBA positive cells (1 week: *p* < 0.001; 4 weeks: *p* < 0.01) and HBB positive cells (1 week: *p* < 0.001; 4 weeks: *p* < 0.01) after 1 week and 4 weeks of UAC stimulation. Similarly, Western blot analysis confirmed a consistent decrease in HBA protein levels (1 week: *p* < 0.01; 4 weeks: *p* < 0.001) and HBB protein levels (1 week: *p* < 0.05; 4 weeks: *p* < 0.001) (Figure 2C, D). These findings indicate that HB expression exhibits a declining trend during the early stage of TMJ OA progression.

### 3.3. HB Expression was Decreased in Chondrocytes Following FFSS Application In Vitro

**Figure 3:**
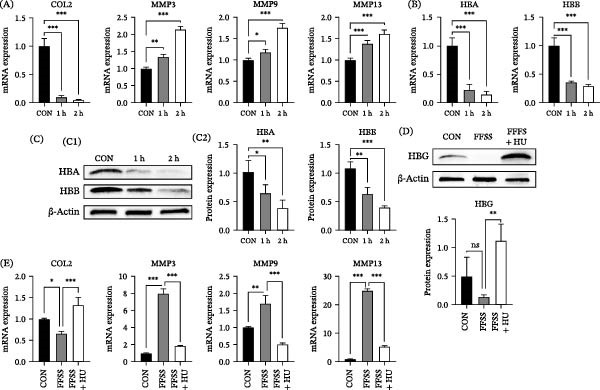
Hydroxyurea (HU) alleviates chondrocyte degeneration induced by fluid flow shear stress (FFSS). (A) Application of 16 dyn/cm^2^ FFSS to ATDC5 cells simulated the OA‐like pathological changes, resulting in decreased *COL2* mRNA expression and increased *MMP3*, *MMP9*, and *MMP13* mRNA expression. (B) FFSS significantly reduced *HBA* and *HBB* mRNA expression. (C) Western blot analysis confirmed decreased HBA and HBB protein levels after FFSS (C1). Quantification of biological replicates is shown (C2; normalized to β‐actin). Full, uncropped Western blot images are provided in Figure S4. (D) HBG protein (~12 kDa) expression decreased after 2 h of 16 dyn/cm^2^ FFSS, but increased with HU (25 μmol/mL) treatment. Quantification data are shown below (normalized to β‐actin). Full, uncropped Western blot images are provided in Figure S5. (E) HU pretreatment increased *COL2* mRNA expression and decreased *MMP3*, *MMP9* and *MMP13* mRNA expression. Data are presented as mean ± SD from three independent experiments (*n* = 3). CON, control; 1 h, 1 hour of FFSS; 2 h, 2 hours of FFSS;  ^∗^ *p* < 0.05;  ^∗∗^ *p* < 0.01;  ^∗∗∗^ *p* < 0.001.

Mouse ATDC5 chondrocytes were cultured under hypoxic conditions (5% O_2_) and then subjected to FFSS (16 dyn/cm^2^) for 1 h or 2 h to establish an in vitro OA model. With prolonged FFSS exposure, *COL2* expression significantly decreased (both time points: *p* < 0.001), whereas *MMP3* (1 h: *p* < 0.01; 2 h: *p* < 0.001), *MMP9* (1 h: *p* < 0.05; 2 h: *p* < 0.001), and *MMP13* (both time points: *p* < 0.001) expression was markedly elevated (Figure 3A). These gene expression alterations indicate disrupted chondrocyte metabolic homeostasis, confirming that FFSS successfully recapitulates OA‐like changes in vitro. Furthermore, FFSS application significantly reduced mRNA expression of *HBA* and *HBB* (both time points: *p* < 0.001) (Figure 3B). Western blot analysis corroborated these findings, showing decreased HBA (1 h: *p* < 0.05; 2 h: *p* < 0.01) and HBB (1 h: *p* < 0.01; 2 h: *p* < 0.001) protein levels (Figure 3C), further supporting diminished HB expression in the FFSS‐induced OA model.

### 3.4. HU Effectively Alleviated OA Progression In Vitro and In Vivo

Compared with the control group, HBG protein expression declined after 2 h of FFSS (16 dyn/cm^2^), but after pretreatment of HU (25 μmol/mL), HBG expression significantly increased (*p* < 0.01) (Figure 3D). Concurrently, *COL2* expression was upregulated (*p* < 0.001), while the expression of *MMP3* (*p* < 0.001), *MMP9* (*p* < 0.001), and *MMP13* (*p* < 0.001) was downregulated. These results indicate that HU treatment alleviated FFSS induced chondrocyte degeneration in vitro (Figure 3E).

**Figure 4:**
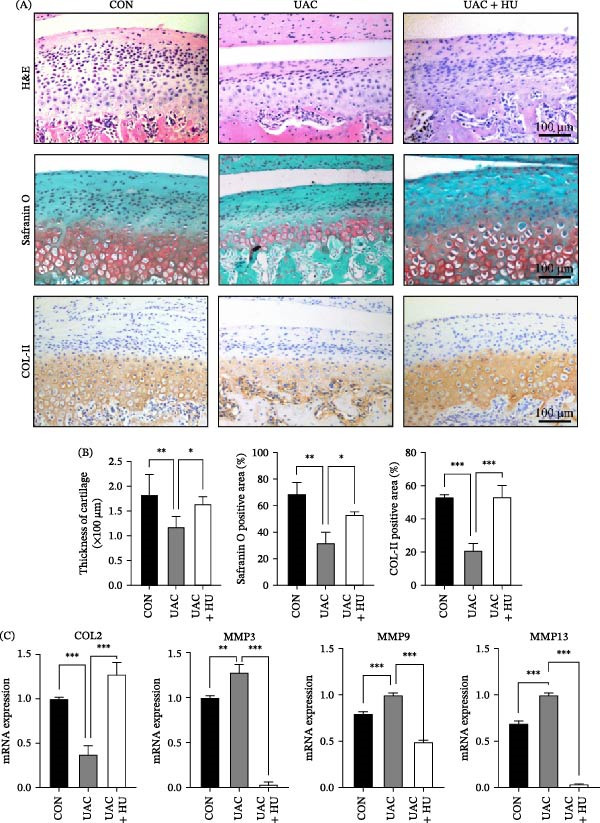
Hydroxyurea (HU) alleviates TMJ OA progression induced by UAC stimulation. (A, B) Histological analysis of TMJ cartilage. H&E staining revealed a significant restoration of cartilage thickness in HU‐treated rats compared with the UAC group. SO staining demonstrated improved proteoglycan content following HU treatment. IHC analysis showed a marked increase in COL II expression in HU‐treated rats. (C) qRT‐PCR results indicated that HU treatment upregulated *COL2* mRNA expression while downregulated *MMP3*, *MMP9*, and *MMP13* in TMJ tissues. Data are presented as mean ± SD (*n* = 6 rats per group). CON, control; UAC, unilateral anterior crossbite; UAC + HU, UAC group treated with HU;  ^∗^ *p* < 0.05;  ^∗∗^ *p* < 0.01;  ^∗∗∗^ *p* < 0.001; scale bars: 100 μm.

To investigate the therapeutic potential of HU for TMJ OA, we administered intra‐articular HU injections to UAC model rats and then collected bilateral TMJ tissues for evaluation. As degenerative changes were evident as early as 1 week after UAC, we aimed to evaluate the therapeutic potential of HU over a longer term (4 weeks) to determine if its effects were sustainable and could ameliorate progressive cartilage degeneration. The results of H&E staining and SO staining on paraffin sections showed that the cartilage layer thickened (*p* < 0.05) and proteoglycan content increased (*p* < 0.05) after HU treatment (Figure 4A, B). Immunohistochemistry staining for COL II revealed enhanced expression of COL II (*p* < 0.001) in the cartilage, indicating that HU treatment ameliorated UAC induced cartilage degeneration. Quantitative real‐time PCR analysis showed that, compared with UAC group, mRNA levels of cartilage matrix synthesis related markers (*COL2*) were upregulated, while those of degradation related markers (*MMP3*, *MMP9*, and *MMP13*) were downregulated (*p* < 0.001). These findings suggest that in vivo HU injection effectively alleviate OA pathology.

## 4. Discussion

In this study, a UAC rat model was established to simulate TMJ OA‐like pathological changes. Histological and molecular analyses revealed a significant decrease in HB expression during the early phase of TMJ OA. These in vivo findings were further validated through in vitro experiments. Intra‐articular administration of HU into the TMJ significantly attenuated UAC induced condylar cartilage degeneration by upregulating chondrocytic HB expression, suggesting that HB may play a crucial role in the pathophysiology of TMJ OA.

HB, a tetrameric heme‐containing protein in red blood cells, binds oxygen in the lungs and releases it during systemic circulation [4, 28]. Traditionally considered erythrocyte‐specific, recent studies have identified ectopic HB expression in non‐erythroid cells. For example, HB is selectively enriched in A9 dopaminergic neurons. While it exerts protective effects such as maintaining mitochondrial homeostasis and regulating iron metabolism, its overexpression induces iron deposition and α‐synuclein aggregation, contributing to Parkinson’s disease pathogenesis [29]. In hepatocytes, HB attenuates ROS‐mediated oxidative stress and protects against peroxidation damage [30]. Notably, Zhang’s research group first identified HB expression in chondrocytes. This finding challenges the conventional understanding of chondrocyte energy metabolism and provides new perspectives on cartilage metabolic regulation and pathophysiology [11].

Articular cartilage, an avascular tissue, relies on nutrient diffusion from the synovial fluid and subchondral bone vasculature. Consequently, chondrocytes reside in a hypoxic microenvironment, but require specialized mechanisms to capture and store oxygen for survival [31]. Zhang’s research elucidated that chondrocytes synthesize membrane‐less HB to store oxygen diffusing from the subchondral bone for short‐distance oxygen transport. This mechanism is essential for chondrocyte oxygenation, viability, and function, offering novel insights into cartilage‐specific oxygen delivery systems [11]. However, the role of HB in OA cartilage remains unclear. An interesting observation from our study was the significant difference in baseline HBA and HBB expression in control cartilage between 1 and 4 weeks. This finding aligns with recent evidence demonstrating that HB expression in chondrocytes is developmentally regulated. Zhang et al. [11] reported that in mice, *HBA* and *HBB* mRNA levels in cartilage declined from postnatal day 7 (P7) to P40, indicating an age‐dependent downregulation of HB production in chondrocytes. In our study, the control rats were 7 weeks old at the 1‐week time point and 10 weeks old at the 4‐week time point, spanning a comparable developmental window. Thus, the observed difference likely reflects a physiological decrease in HB expression as the condylar cartilage matures.

HU, a potent inhibitor of ribonucleotide reductase, suppresses nucleic acid synthesis and is clinically used to treat hematological malignancies such as chronic myelogenous leukemia and polycythemia vera [32, 33]. By inhibiting ribonucleotide reductase, HU effectively suppresses neoplastic hematopoietic cell proliferation, thereby exerting its therapeutic effects in these blood‐related tumor conditions. This mechanism of action makes it an important therapeutic option in the comprehensive treatment strategies for such hematological disorders, contributing to disease progression control, symptom alleviation, and improved patient quality of life and prognosis [34–36]. Recent studies demonstrate its capacity to induce the production of HBF with high oxygen affinity, which enhances tissue oxygenation and improves hypoxic adaptation [37]. HBF, composed of two α‐ and two γ‐globin chains (α2γ2), is predominantly expressed in embryos and newborns. Postnatally, through physiological transitions associated with spontaneous respiration, HBF is gradually replaced by adult HB (HBA). However, HBF exhibits a higher oxygen‐binding affinity than HBA [18, 38]. Clinically, HU has been used to treat sickle cell anemia by elevating HBF levels, thereby ameliorating hypoxia in patients [39, 40].

While our study demonstrates the chondroprotective effects of intra‐articular HU, it is important to acknowledge its potential systemic side effects, such as myelosuppression, when used clinically [41, 42]. Although HU has a short half‐life in rodents (~15–90 min depending on the compartment [43]), its pharmacodynamic effect on HBF induction is prolonged, supporting the twice‐weekly dosing regimen used in this study [23, 24]. Recent studies have further elucidated the molecular mechanisms underlying HBF induction, demonstrating that HU modulates γ‐globin expression through pathways involving IGF2BP1 and GCNT2 in erythroid cells [44]. Notably, the local intra‐articular administration used in our study may minimize systemic risks; however, future studies should assess the local safety profile of this delivery method, including potential effects on synovial tissue and cartilage.

A major limitation of this study is that HU possesses well‐documented anti‐inflammatory properties independent of its effect on HB induction. Studies have shown that HU can modulate inflammatory responses through nitric oxide‐dependent pathways [45] and affect cytokine production in various cell types. Therefore, the observed chondroprotective effects in vivo could be partially attributed to this anti‐inflammatory activity. While our in vitro data suggest that HU can upregulate HBG expression in chondrocytes under FFSS (Figure 3D), and recent studies have confirmed its direct effect on γ‐globin expression via IGF2BP1 and GCNT2 [44], we cannot definitively dissociate the direct effects of increased HB from the indirect effects of reduced inflammation in the complex in vivo UAC model. Future studies using genetic approaches, such as conditional overexpression of *HBB* or *HBA* specifically in chondrocytes, are necessary to confirm the direct causal role of HB in cartilage protection and to distinguish it from the drug’s pleiotropic effects.

In addition, only female rats were used in this study. This decision was based on the well‐established female predilection of TMDs in humans [20] and to avoid sex‐related hormonal variations in this exploratory study. However, the estrus cycle was not monitored, which represents a limitation as hormonal fluctuations could potentially influence cartilage metabolism and inflammatory responses. Future studies should include both male and female animals with careful monitoring of estrus cycles to validate the generalizability of our findings and to explore potential sex‐dependent effects of HU treatment.

## 5. Conclusions

In summary, TMJ OA‐like cartilage degeneration was successfully induced by UAC stimulation. This study revealed a significant reduction in HB expression in condylar cartilage during early‐stage TMJ OA progression. Notably, HU administration effectively attenuated cartilage degeneration by restoring HB levels, demonstrating its potential therapeutic efficacy.

## Author Contributions

**Min Hui:** conceptualization, data curation, formal analysis, investigation, writing – original draft, writing – review and editing. **Zhihua Xu, Qinghua Li, Ying Zhan, Yuqian Shi, and Fan Wu:** conceptualization, data curation, formal analysis, writing – review and editing. **Lei Lu, and Mian Zhang:** conceptualization, methodology, writing – review and editing. **Hongxu Yang:** methodology, writing – review and editing, funding acquisition. **Shibin Yu:** conceptualization, methodology, data curation, formal analysis, writing – review and editing, funding acquisition. **Jing Zhang:** conceptualization, methodology, data curation, formal analysis, writing – original draft, writing – review and editing, supervision, funding acquisition.

## Funding

This study was supported by the National Natural Science Foundation of China (Grants 82571120, 82271000, and 82471001) and the National Natural Science Foundation of Shaanxi (Grant 2024ZC‐KJXX‐123).

## Disclosure

All authors gave their final approval and agree to be accountable for all aspects of the work.

## Conflicts of Interest

The authors declare no conflicts of interest.

## Supporting Information

Additional supporting information can be found online in the Supporting Information section.

## Supporting information


**Supporting Information** Table S1 provides the primer sequences employed in the experimental procedures. Figure S1 CCK‐8 assay of the viability of ATDC5 cells treated with increasing concentrations of hydroxyurea. Figures S2–S5 showing the full‐length Western blot images for HBA, HBB, and β‐actin.

## Data Availability

The data that support the findings of this study are available from the corresponding author upon reasonable request.
