# Supplementary material for: The Role of Hemoglobin in Temporomandibular Joint Osteoarthritis and the Therapeutic Potential of Hydroxyurea
Source: Int J Dent. 2026 May 19;2026:3607127. doi: 10.1155/ijod/3607127 (PMC13184826; doi:10.1155/ijod/3607127)
Supplement: Supplementary file 1 — Supporting Information Table S1 provides the primer sequences employed in the experimental procedures. Figure S1 CCK‐8 assay of the viability of ATDC5 cells treated with increasing concentrations of hydroxyurea. Figures S2–S5 showing the full‐length Western blot images for HBA, HBB, and β‐actin. [file IJOD-2026-3607127-s001.docx]

| Genes | | Rat | Mouse |
| --- | --- | --- | --- |
| *COL2* | F | GGAGCAGCAAGAGCAAGGAGAAG | ACGCTACACTCAAGTCACTGAACAAC |
|  | R | TCAGTGGACAGTAGACGGAGGAAAG | TCAATCCAGTAGTCTCCGCTCTTCC |
| *MMP3* | F | TGATGATGAACGATGGACAGATGATG | GACGATGATGAACGATGGACAGAG |
|  | R | GCATTGGCTGAGTGAAAGAGACC | GCCTTGGCTGAGTGGTAGAGTC |
| *MMP9* | F | CTCCTGGTGCTCCTGGCTCTAG | AATAAAGACGACATAGACGGCATCC |
|  | R | GCTGTGTGTCCGTGAGGTTGG | AGTTGTGGTGGTGGCTGGAG |
| *MMP13* | F | CCTTCCCTATGGTGATGATGATGATG | CAGTTGACAGGCTCCGAGAAATG |
|  | R | GCAAGAGTCACAGGATGGTAGTATG | CACATCAGGCACTCCACATCTTG |
| *HBA* | F | CATGGTGGTGAATATGGCGAGGAG | CGGTATTTGGAGGTCAGCAC |
|  | R | CCTTCTTGCCGTGAGCCTTGAC | GACCCGGTCAACTTCAAGC |
| *HBB* | F | TGTCCTCTGCCTCTGCTATCATGG | AGGTGAACGCCGATGAAGTT |
|  | R | ATGCAGCTTGTCACAGTGGAGTTC | ATGCAGCTTGTCACAGTGGA |

Supplemental Table 1. Primer Sequences

Supplemental Figure 1


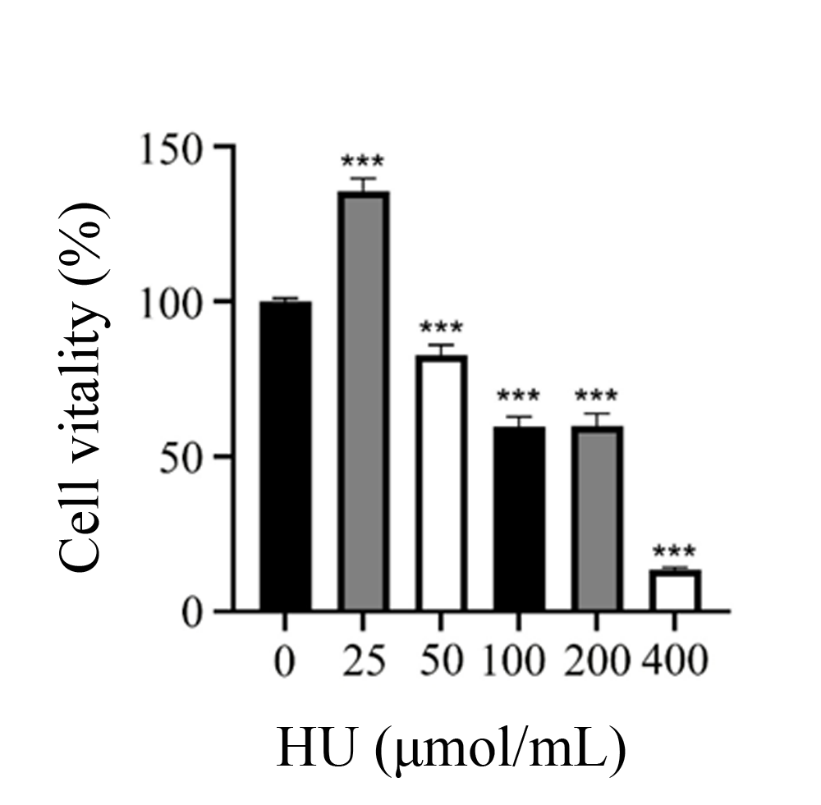


**Fig. S1** CCK-8 assay of the viability of ATDC5 cells treated with increasing concentrations of hydroxyurea (HU) for 24 h. Data are presented as mean ± SD (n = 3). ***, p < 0.001 vs. control group (0 μmol/mL).

Supplemental Figure 2


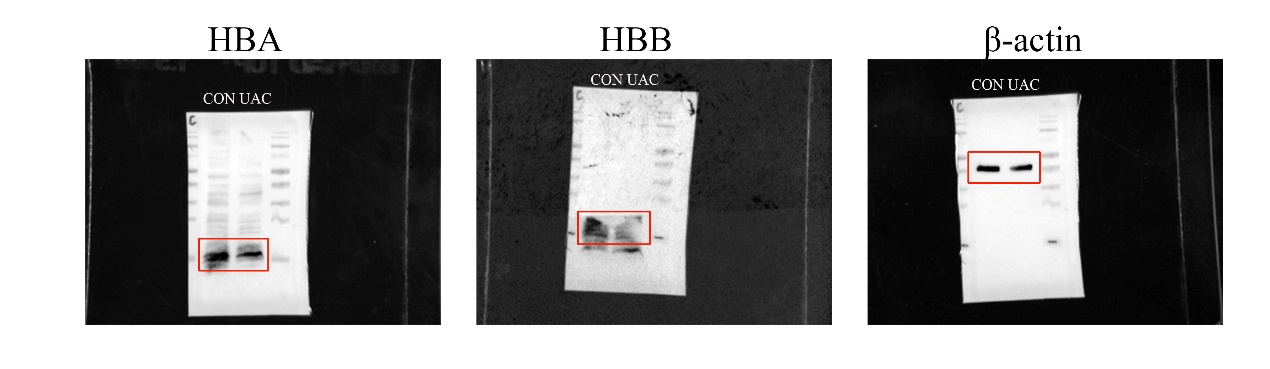


**Fig. S2** Full-length Western blot images for HBA, HBB, and β-actin in 1-week control (CON) and unilateral anterior crossbite (UAC) groups. Molecular weight markers: HBA ~15 kDa, HBB ~16 kDa, β-actin ~42 kDa. The regions corresponding to the cropped images presented in Figure 2C are indicated by boxes.

Supplemental Figure 3


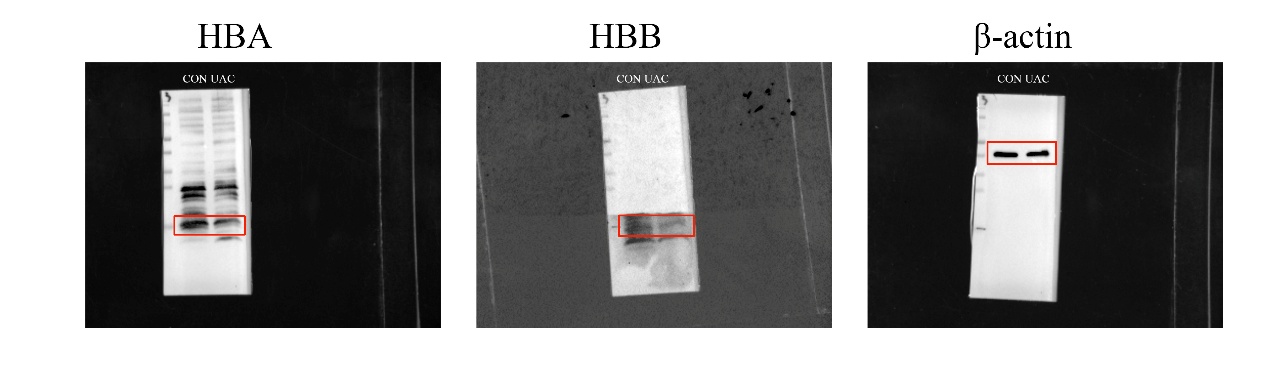


**Fig** **S3** Full-length Western blot images for HBA, HBB, and β-actin in 4-week control (CON) and unilateral anterior crossbite (UAC) groups. Molecular weight markers: HBA ~15 kDa, HBB ~16 kDa, β-actin ~42 kDa. The regions corresponding to the cropped images presented in Figure 2C are indicated by boxes.

Supplemental Figure 4


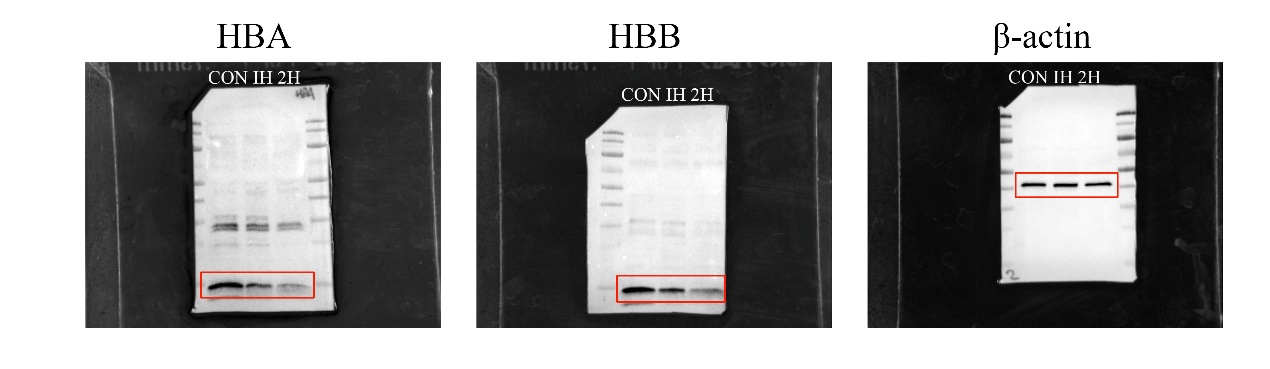


**Fig. S4** Full-length Western blot images for HBA and HBB after fluid flow shear stress (FFSS) application. Molecular weight markers: HBA ~15 kDa, HBB ~16 kDa, β-actin ~42 kDa. The regions corresponding to the cropped images presented in Figure 3C are indicated by boxes. CON, control; 1 H, 1 hour of FFSS; 2 H, 2 hours of FFSS.

Supplemental Figure 5


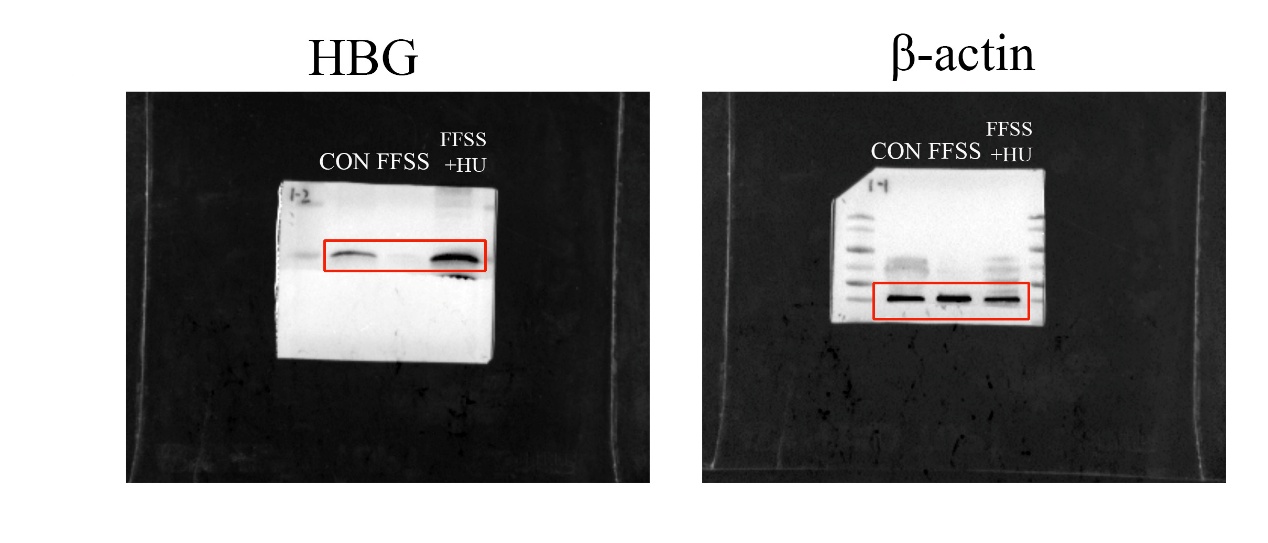


**Fig. S5** Full-length Western blot images for HBG after FFSS application with or without hydroxyurea treatment. Molecular weight markers: HBG ~12 kDa, β-actin ~42 kDa. The regions corresponding to the cropped images presented in Figure 3D are indicated by boxes. CON, control; FFSS, fluid flow shear stress; HU, hydroxyurea; 1 H, 1 hour of FFSS; 2 H, 2 hours of FFSS.
